# Supplementary material for: Complex I preserves mitochondrial polarization during infection of human macrophages by secretion-competent bacteria
Source: Infect Immun. 2026 Feb 27;94(4):e00495-25. doi: 10.1128/iai.00495-25 (PMC13081716; doi:10.1128/iai.00495-25)
Supplement: Fig. S1 — Related to Figure 2, 3 and 4. [file iai.00495-25-s0001.pdf]

Supplementary Information

Article: **Complex-I Preserves Mitochondrial Polarization during Infection of Human Macrophages by Secretion-competent Bacteria**, by Francisco-Javier Garcia-Rodriguez, Paula Martinez-Oca, Carmen Buchrieser and Pedro Escoll

Supplementary Figure

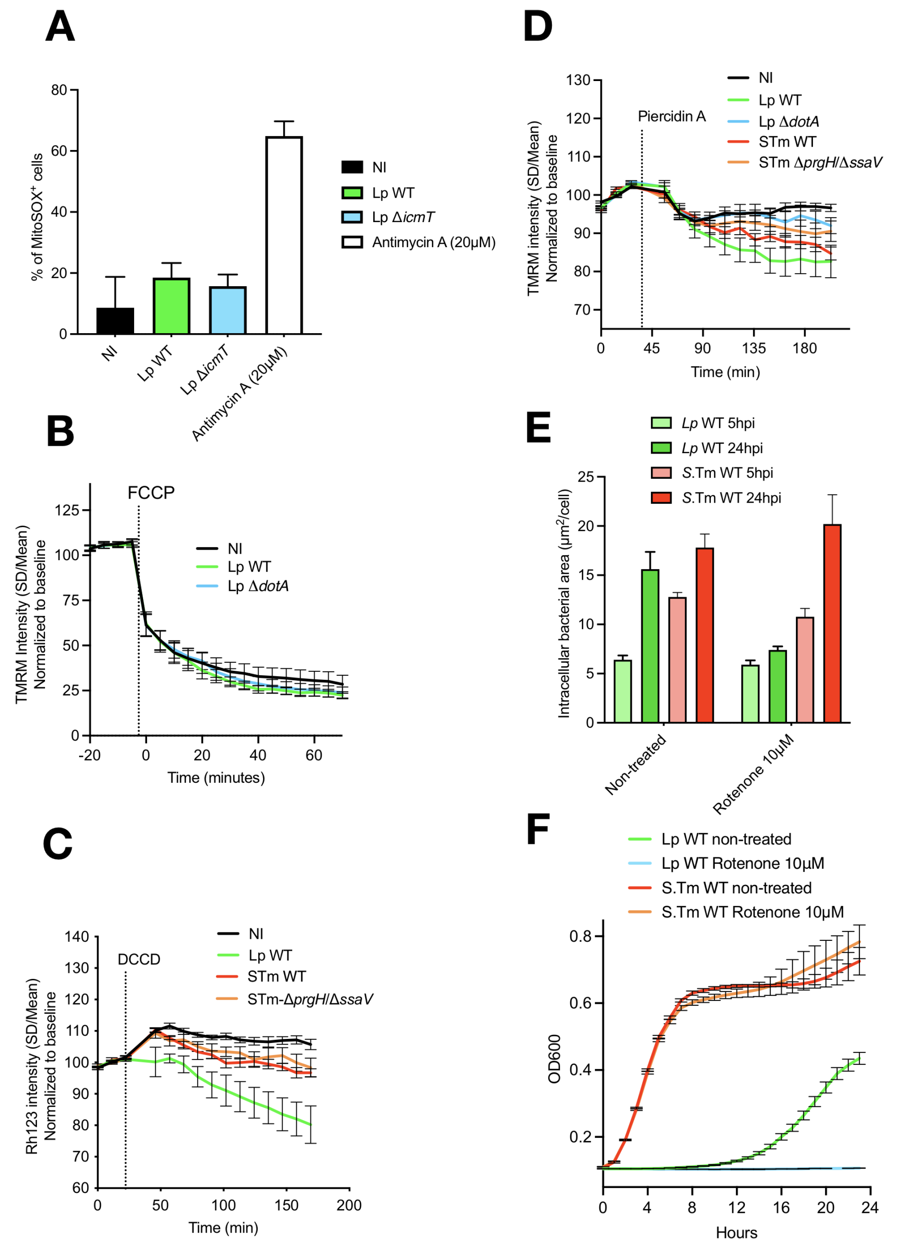

Figure S1. Related to Figure 2, 3 and 4. (**A**) Percentage of MitoSOX$^+$ hMDMs at 5 hpi, measured by flow cytometry. Human macrophages were kept uninfected (NI=non-infected), infected with *Lp*-WT, with *Lp*-Δ*icmT* (a T4SS-deficient mutant), or challenged with Antimycin A (positive control). Bars represent the mean ± SD of three independent experiments from two different donors. (**B**) Δψm (TMRM) after addition of FCCP at 5 hpi in NI, *Lp*-WT, and *Lp*-Δ*dotA*-infected hMDMs. (**C**) Same as Figure 3B and 3C but using Rhodamine 123 as probe to measure Δψm. (**D**) Same as Figure 4B but using Piericidin A (10 µM) to inhibit ETC Complex I. (**E**) Area occupied by intracellular bacteria, *Lp*-WT or *S*.Tm-WT, within infected hMDMs at 5 and 24hpi in non-treated or Rotenone-treated conditions (measured in µm^2 per cell). (**F**) Axenic growth of *Lp*-WT and *S*.Tm-WT in non-treated or Rotenone-treated conditions (measured by continuous monitoring of OD_{600})
